# Organocatalytic Michael Addition to (D)-Mannitol-Derived Enantiopure Nitroalkenes: A Valuable Strategy for the Synthesis of Densely Functionalized Chiral Molecules

**DOI:** 10.3390/molecules24244588

**Published:** 2019-12-14

**Authors:** Lucia Caruso, Alessandra Puglisi, Emmerance Gillon, Maurizio Benaglia

**Affiliations:** Dipartimento di Chimica, Università degli Studi di Milano, Via Golgi 19, 20133 Milano, Italy; lucia.caruso@unimi.it (L.C.); alessandra.puglisi@unimi.it (A.P.); emmerance.gillon@student.uclouvain.be (E.G.)

**Keywords:** nitro alkenes, stereoselective synthesis, renewable sources, organocatalysis, chiral molecules

## Abstract

Carbohydrates are abundant renewable resources and are a feedstock for green chemistry and sustainable synthesis of the future. Among the hexoses and the pentoses present in biomass, mannitol was selected in the present project as a valuable platform, directly available from the chiral pool, to build highly functionalized molecules. Starting from (*R*)-2,3-O-cyclohexylidene glyceraldehyde, which is easily prepared in a large scale from D-mannitol, an enantiopure chiral nitro alkene was prepared by reaction with nitromethane, and its reactivity studied. Organocatalytic Michael addition of dimethyl malonate, β-keto esters, and other nucleophiles on the nitro alkene afforded high stereoselectivity and densely functionalized chiral molecules, which were further synthetically developed, leading to five-membered lactones and bicyclic lactams. Preliminary studies showed that the metal-free catalytic reaction on the chiral nitro alkene can be performed under continuous flow conditions, thus enabling the use of (micro)mesofluidic systems for the preparation of enantiomerically pure organic molecules from the chiral pool.

## 1. Introduction

Efficient use of resources is the key to realizing sustainable growth through the development of new technologies with low environmental impact [1]. This will also lead to the creation of new jobs [2]. In this framework, one of the challenges is the replacement of nonrenewable carbon sources, such as oil, by alternative renewable sources, including biomass, which is gaining increasing attention as a source of energy [3].

New chemistry has to be developed in order to convert natural starting materials into a variety of chemicals comparable to the oil-derived ones. Such an effort asks for fundamental research covering a novel series of synthetic transformations from bio-based building blocks. For instance, rather than the functionalization of hydrocarbon-derived intermediates, the defunctionalization of easily available and redundantly functionalized natural building blocks will become critical. An innovative series of fine chemicals, endowed with similar or improved properties (e.g., better biodegradability), will emerge from these studies [4].

Seventy-five percent of biomass is carbohydrates and only 3–4% is used by industry. Sugars are the most abundant renewable resources and they are seen as the feedstock for green chemistry of the future. [5]

In this work, we selected 1,2:5,6-Di-O-cyclohexylidene-protected D-mannitol as a valuable enantiopure starting material for the preparation of chiral compounds [6]. D-mannitol has found extensive use in organic synthesis and it been employed for the synthesis of chiral ligands [7], organocatalysts [8], and in total synthesis [9]. In our study, we exploited D-mannitol for the synthesis of an enantiomerically pure nitro alkene. Such compounds have recently attracted a lot of attention due to their synthetic versatility: nitroalkanes are direct precursors of amines [10] and may be used as nucleophiles, while nitro alkenes are excellent electrophilic reagents and reactive dienophiles in cycloaddition reactions; the role of the nitro group as a hydrogen bond acceptor to guarantee an efficient mutual reagent/catalyst recognition has been extensively exploited in several organocatalytic transformations [11].

**Scheme 1:**
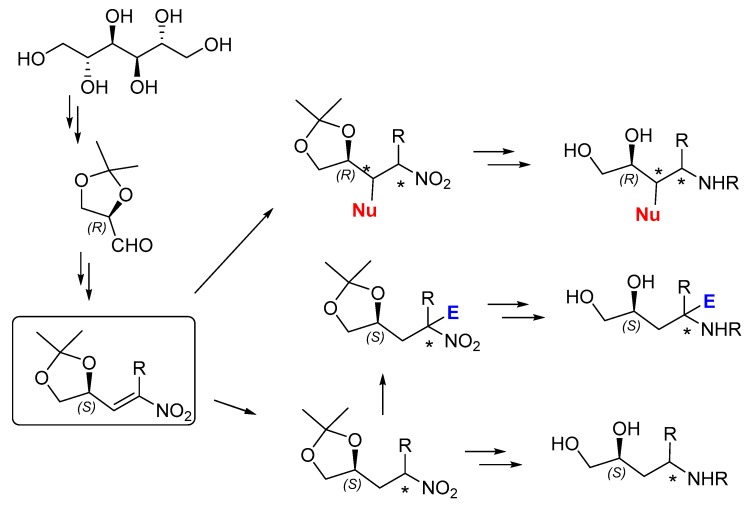
D-Mannitol-derived chiral nitro alkenes and their reactivity.

Starting from (*R*)-2,3-O-cyclohexylidene glyceraldehyde, easily prepared on a large scale from D-mannitol, enantiopure chiral nitro alkenes may be obtained by reaction with nitroalkanes, and their reactivity explored to synthesize functionalized molecules featuring tertiary and quaternary stereocenters, as seen in Scheme 1.

## 2. Results and Discussion

**Scheme 2:**
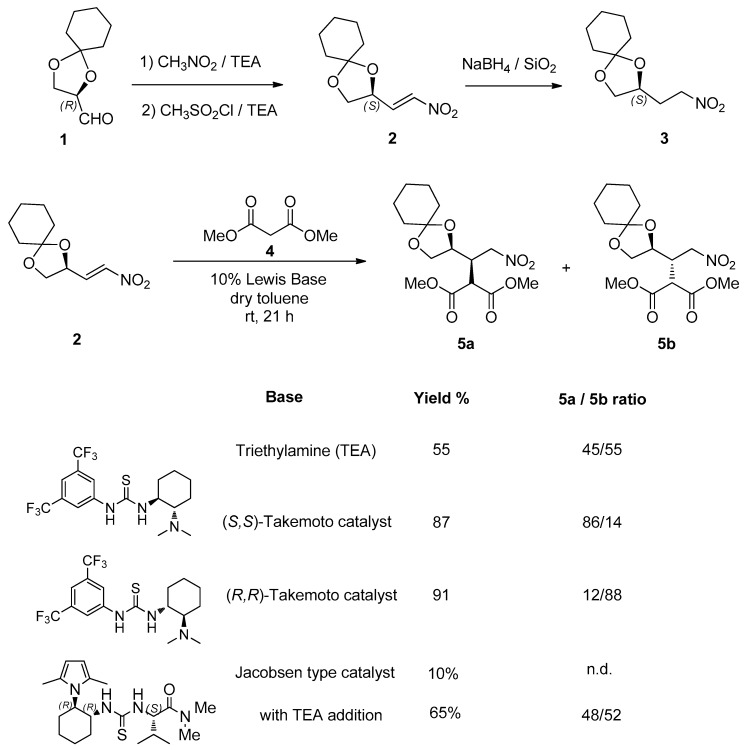
Stereoselective catalytic Michael malonate addition to nitroalkene **2.**

This article reports the catalytic Michael addition of dimethyl malonate and methyl phenylacetylacetate to the known sugar nitro olefin **2**, which was obtained from (*R*)-2,3-O-cyclohexylidene glyceraldehyde **1** following a known procedure (see experimental section). The reduction of the carbon–carbon double bond to the corresponding nitroalkane **3** was also accomplished with sodium borohydride and silica in a nonoptimized 55% yield, as seen in Scheme 2.

We started our investigation on the reactivity of nitro olefin **2** by studying the catalytic Michael addition of dimethyl malonate **4** in the presence of an achiral base (triethylamine, TEA). A diastereomeric mixture **5a**–**5b** resulted (45/55 ratio, determined by H-NMR), confirming that the malonate addition is poorly stereocontrolled by the stereocenter of the starting compound **2**, as seen in Scheme 2.

In order to improve the diastereoselectivity, a catalytic version of this Michael addition was assayed, using Takemoto’s catalyst [12]. The reaction promoted by the (*S*,*S*)-catalyst afforded the expected mixture **5a**–**5b** in high yield (87%) and 86/14 isomeric ratio. When the (*R*,*R*) enantiomer of the catalyst was used, the Michael adduct mixture **5a**–**5b** was isolated in 91% yield and 12/88 ratio, with opposite diastereofacial selectivity. The absolute configuration of the two isomers was assigned based on the stereofacial preference of Takemoto catalyst, as reported in the literature and taking into account that the configuration of the nitroalkene is (*S*).

Other thiourea-based catalysts, such as Jacobsen-type catalysts, were less active, highlighting the need of a basic site in the bifunctional catalyst in order to efficiently promote the Michael addition to such nitroalkenes.

**Scheme 3:**
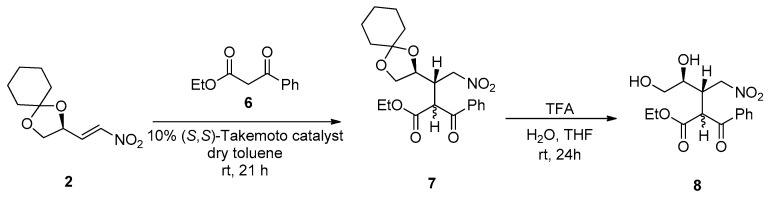
Stereoselective catalytic Michael addition of β-keto ester **6** to nitroalkene **2**.

The reaction between the β-ketoester **6** and nitroalkene **2** was also preliminarily investigated; either with TEA or the chiral Takemoto catalyst, the reaction afforded the adduct **7** in good yields (51% and 70%, respectively) as a mixture of isomers; however, due to the presence of an additional stereocenter, it was not possible to unambiguously determine the isomeric ratio, as seen in Scheme 3. Deprotection of the diol in the presence of water and trifluoroacetic acid [13] led to the formation of compound **8**, which is currently under investigation as a starting material for several transformations (hydrolysis, decarboxylation, nitro reduction, intramolecular reductive amination).

**Scheme 4:**
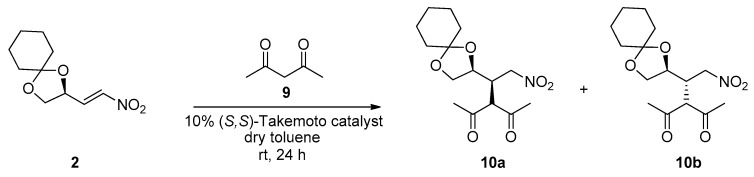
Stereoselective catalytic Michael addition of acetylacetone **9** to nitroalkene **2**.

Beyond the β-ketoester **6**, we also studied the addition of the diketone acetylacetone **9** to the nitroalkene **2** in presence of the same (*S*,*S*)-Takemoto catalyst and the reaction worked quite well (73% yield), as seen in Scheme 4. We obtained the diastereoisomers **10a**,**b** in 82/18 ratio, which is comparable to that reached in the reaction shown in Scheme 2. 

Finally, further reactions were studied for the catalytic Michael addition reaction on the same substrate **2** by employing nitromethane as the nucleophile, but under the present experimental conditions, only a very small amount of addition product was detected and the starting material was almost quantitatively recovered. However, by adding a stoichiometric amount of triethylamine, the reaction produced a 70% nonoptimized yield, but with only very moderate diastereoselectivity. Further studies are needed with other catalysts in order to address the issue of the observed lack of reactivity of nitroalkanes.

**Scheme 5:**
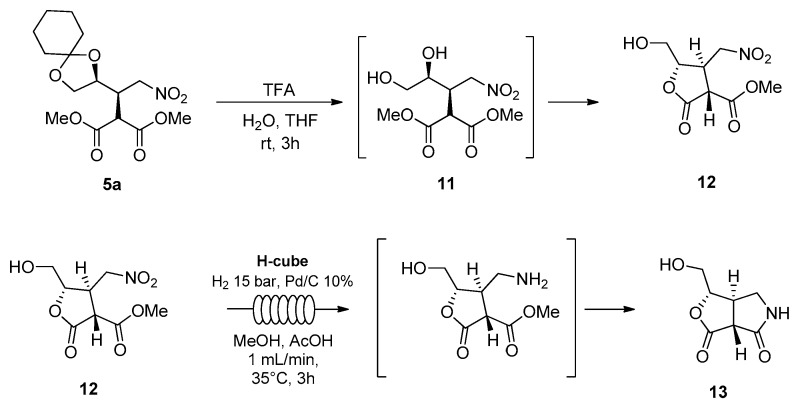
Synthetic elaboration of adduct **5** to lactone **12** and lactam **13**.

Synthetic elaboration of adduct **5** was more successful and led to the formation of valuable, enantiopure cyclic derivatives, as seen in Scheme 5. 

Starting from isomer **5a**, the reaction with aqueous trifluoroacetic acid directly afforded enantiopure lactone **12** in 65% yield. The structural characterization was performed by ESI mass spectrometry, ^1^H NMR, ^13^C NMR, ^1^H-^13^C HSQC, and ^1^H-^1^H COSY.

The reduction of the nitro group of lactone **12** was accomplished by catalytic hydrogenation, by performing the reaction in methanol and in the presence of acetic acid under continuous flow conditions [14]. A ThalesNano H-Cube^®^ mini was used in recirculating mode, with a cartridge of Pd/C (10%) catalyst, operating at hydrogen pressure of 15 bar, a flow rate of 1 mL/min, and a stable temperature of 35 °C. The reaction was monitored by TLC and showed complete conversion after 3 h. It was observed that the reduction was followed by a spontaneous lactamization to afford the five-membered bicyclic lactam **13**. The relative configuration was confirmed by NMR analysis.

Considering the rising interest in the in-flow preparation of APIs [15,16,17], based on these results and with the aim to further accelerate the reaction, we explored the possibility of developing a continuous flow method for the organocatalytic Michael addition to nitroalkene **2** [18].

**Scheme 6:**
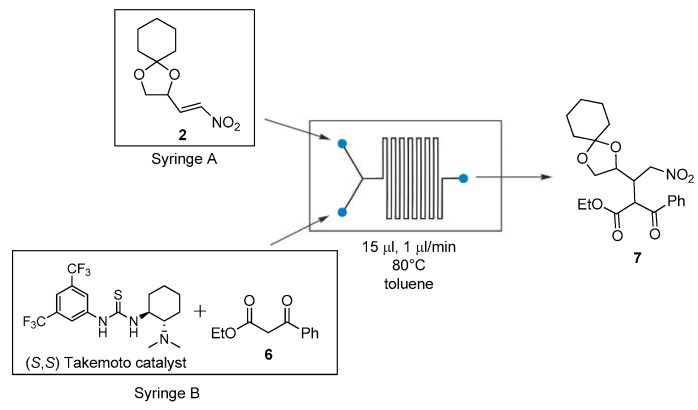
Continuous flow organocatalytic β-ketoester 6 addition to nitroalkene 2 in a microreactor.

Therefore, the Michael addition of the dimethylmalonate to nitroalkene **2** was studied in flow conditions using a Labtrix^®^ Start by Chemtrix provided with a 15 μL glass microreactor, heated at 80 °C, as seen in Scheme 6. A 1 M nitroalkene solution in toluene in syringe A and the dimethyl malonate and Takemoto’s catalyst solution of syringe B were pumped into the reactor. A total flow rate of 1 μL/min and a residency time of 15 min in the reactor were obtained. Under the present conditions, the Michael adduct was isolated in very low yield (<10%). However, better results were observed in the reaction of the β*-*ketoester **6**; operating at the same experimental conditions, product **7** was isolated in 45% yield. Further work is needed to optimize the reaction conditions and to find the correct parameters to successfully perform the malonate addition to nitroalkene, for example, by introducing a premixing phase of the catalyst and the reagents. Although very preliminary, the present results show that the use of microfluidic devices is indeed viable in the synthesis of highly functionalized chiral molecules. 

## 3. Materials and Methods 

Reactions were monitored by analytical thin-layer chromatography (TLC) using silica gel 60 F_254_ precoated glass plates (0.25 mm thickness) and visualized using UV light. Most of the products are UV-visible; KMnO_4_ solution was used to stain the TLC plates and detect compounds **4, 9**, and **13**. Flash chromatography was carried out on silica gel (230–400 mesh). Proton NMR spectra were recorded on spectrometers operating at 300 MHz (Bruker Fourier 300); proton chemical shifts are reported in ppm (δ) with the solvent reference relative to tetramethylsilane (TMS) employed as the internal standard (CDCl_3_ δ = 7.26 ppm). ^13^C-NMR spectra were recorded on 300 MHz spectrometers (Bruker Fourier 300) operating at 75 MHz, with complete proton decoupling; carbon chemical shifts are reported in ppm (δ) relative to TMS with the respective solvent resonance as the internal standard (CDCl_3_ δ = 77.0 ppm). Mass spectra and accurate mass analysis were carried out on a VG AUTOSPEC-M246 spectrometer (double-focusing magnetic sector instrument with EBE geometry) equipped with EI source or with LCQ Fleet ion trap mass spectrometer, ESI source, with acquisition in positive ionization mode in the mass range of 50–2000 *m*/*z*. For the flow experiments, a Chemtrix Labtrix^®^ Start Standard platform equipped with a Chemyx Fusion 100 syringe pump and two Hamilton gastight syringes was used. 

### 3.1. General Procedure for the Synthesis of (R)-Cyclohexylideneglyceraldehyde ***1***

The 1,2:5,6-Di-O-cyclohexylidene-D-mannitol (2.00 g, 5.85 mmol) and NaIO_4_ (1.25 g, 5.86 mmol) were added in 13 mL of H_2_O and 25 mL of EtOAc. The solution was stirred for 3 h at 35 °C [19]. The reaction mixture was extracted with 3 *×* 20 mL EtOAc and dried over Na_2_SO_4_. After evaporation under reduced pressure, the combined organic filtrates were carefully concentrated in vacuo and the crude mixture was purified by distillation under reduced pressure (approximately 20 mbar) and at a temperature of 90 °C, to yield a colorless oil as product (1.99 g, 99%). ^1^H NMR (300 MHz, CDCl_3_) δ 9.73 (d, J = 1.7 Hz, 1H), 4.39 (m, 1H), 4.17 (dd, J = 8.8, 7.3 Hz, 1H), 4.10 (dd, J = 8.8, 4.8 Hz, 1H), 1.72–1.53 (m, 8H), 1.49–1.37 (m, 2H).

### 3.2. General Procedure for the Synthesis of (E)-2-(2-Nitrovinyl)-1,4-Dioxaspiro [4.5] Decane ***2***

TEA (0.270 mL, 1.936 mmol) was added to an ice cooled solution of nitromethane (2.00 mL, 37.3 mmol). After 15 min, (R)-Cyclohexylideneglyceraldehyde **1** (1.99 g, 11.7 mmol) dissolved in 9.00 mL of nitromethane was added dropwise over a period of 15 min. The reaction mixture was allowed to reach room temperature (RT) and stirred for 21 h. The excess nitromethane was removed under reduced pressure. The crude mixture is then purified by column chromatography on silica gel (hexane/EtOAc = 3/7 as eluent, KMnO_4_ stained) [20]. After removing the solvents under reduced pressure, a yellow oil was obtained (2.55 g, 94%). The nitro aldols are afforded as diastereoisomeric ratio 1/10. ^1^H NMR (300 MHz, CDCl_3_) δ 4.78 (dd, J = 7.2, 2.2 Hz, 1H, minor), 4.70 (dd, J = 13.9, 2.3 Hz, 1H, major), 4.66–4.51 (m, 1H, minor), 4.54–4.40 (m, 1H, major), 4.38–4.30 (m, 1H, minor), 4.25–4.17 (m, 1H, major), 4.17–4.08 (m, 2H, both isomers), 4.05–3.92 (m, 4H, both isomers), 1.66–1.49 (m, 16H, both isomers), 1.45–1.33 (m, 4H, both isomers). To a solution of 2-nitro-1-(1,4-dioxaspiro[4.5]decan-2-yl)ethanol (2.55 g, 11.0 mmol) in 5.50 mL of dry DCM at −30 °C was added MsCl (1.80 mL, 23.3 mmol), followed by the addition of TEA (3.40 mL, 24.4 mmol) over a period of 30 min. After 15 min H_2_O was added to the reaction mixture and the organic phase was extracted with 3 × DCM, washed with brine and dried over Na_2_SO_4_. The solvents were removed under reduced pressure. The crude mixture was then purified by column chromatography on silica gel (hexane/EtOAc = 8/2 as eluent, KMnO_4_ stained). After evaporation of the solvents under reduced pressure, a yellow oil was obtained (1.04 g, 44%); ^1^H NMR (300 MHz, CDCl_3_) δ 7.21 (s, 2H), 4.79 (td, J = 6.7, 2.1 Hz, 1H), 4.26 (dd, J = 8.4, 6.8 Hz, 1H), 3.76 (dd, J = 8.4, 6.6 Hz, 1H), 1.71–1.54 (m, 8H), 1.47–1.36 (m, 2H) [21].

### 3.3. General Procedure for the Synthesis of 2-(2-Nitroethyl)-1,4-Dioxaspiro[4.5]decane ***3***

To a stirred mixture of the nitro alkene (0.215 g, 1.01 mmol), silica gel (2.01 g), 3 mL of i-PrOH, 16 mL of chloroform was added NaBH_4_ (0.173 g, 4.56 mmol) in 40.0 mg portions over a period of 15 min at RT. The mixture was stirred for additional 15 min. Excess NaBH_4_ was decomposed with dilute aqueous HCl solution and the mixture was filtered. The filter was washed with DCM and the combined filtrates were washed with brine, dried over Na_2_SO_4_, and then evaporated in vacuo to dryness to give the nitro alkane as an orange oil (0.0763 g, 35%). ^1^H NMR (300 MHz, CDCl_3_) δ 4.54 (t, J = 7.4 Hz, 2H), 4.23–4.13 (m, 1H), 4.09 (dd, J = 8.1, 6.1 Hz, 1H), 3.60 (dd, J = 8.1, 6.0 Hz, 1H), 2.32 (m, 1H), 2.20–2.03 (m, 1H), 1.63–1.51 (m, 8H), 1.44–1.32 (m, 2H).

### 3.4. General Procedure for the Synthesis of Dimethyl 2-(2-nitro-1-((S)-1,4-Dioxaspiro[4.5]Decan-2-yl)Ethyl)Malonate ***5a/5b***

To a stirred solution of dimethyl malonate (600 µL, 5.24 mmol) and Takemoto catalyst (0.109 g, 10% mol, 0.264 mmol) in 5 mL of dry toluene was added nitro alkene (0.557 g, 2.61 mmol) at RT. After being stirred for 24 h, the reaction mixture was concentrated in vacuo. The thiourea catalyst was eliminated through a short silica pad (DCM as eluent) and the excess of dimethyl malonate was removed by heating the flask under reduced pressure. An orange oil was obtained (KMnO_4_ stained, 0.901 g, 86%). ^1^H NMR (300 Mhz, CDCl_3_) δ 4.76 (dd, J = 14.8, 5.7 Hz, 1H), 4.67 (dd, J = 14.8, 5.0 Hz, 1H), 4.28 (q, J = 6.3 Hz, 1H), 4.11 (dd, J = 8.7, 6.5 Hz, 1H), 3.77 (s, 3H), 3.75 (s, 3H), 3.72 (dd, J = 8.7, 6.1 Hz,1H), 3.62 (d, J = 5.2 Hz, 1H), 3.18 (J = 5.3 Hz, 1H), 1.62–1.46 (m, 8H), 1.42–1.30 (m, 2H). ^13^C NMR (300 Mhz, CDCl_3_) δ 168.35, 168.04, 110.08, 74.41, 74.15, 66.59, 52.82, 52.48, 49.68, 39.97, 35.71, 34.21, 24.91, 23.78, 23.54; HRMS (ESI+) requires 345.1424, found 345.14104.

### 3.5. General Procedure for the Synthesis of Ethyl 2-benzoyl-4-nitro-3-((S)-1,4-dioxaspiro[4.5]decan-2-yl)butanoate ***7***

To a stirred solution of ethyl 3-oxo-3-phenylpropanoate (1.30 mL, 6.52 mmol) and Takemoto catalyst (0.135 g, 10% mol, 0.326 mmol) in 6.58 mL of dry toluene was added nitro alkene (0.6952 g, 3.26 mmol) at RT. After being stirred for 24 h, the reaction mixture was concentrated in vacuo. Then the reaction mixture was purified by flash column chromatography on silica gel (eluent: hexane*/*AcOEt 8/3) obtaining a yellow oil (1.13 g, 85%). ^1^H NMR (300 MHz, CDCl_3_) δ 8.02 (dd, 1H), 7.76–7.41 (m, 4H), 5.09–4.63 (m, 2H), 4.35–3.98 (m, 4H), 3.74–3.60 (m, 2H), 3.31 (m, 1H), 1.68–1.41 (m, 10H), 1.27–1.13 (m, 3H). ^13^C NMR (300 MHz, CDCl_3_) δ 194.52, 168.20, 136.42, 135.50, 134.03, 133.93, 128.89, 111.29, 75.21, 74.72, 67.04, 62.16, 51.74, 40.89, 34.53, 24.97, 23.83, 13.82. HRMS (ESI+) requires 405.1788, found 405.17742.

### 3.6. General Procedure for the Synthesis of (4S)-ethyl 2-benzoyl-4,5-dihydroxy-3-(nitromethyl)pentanoate ***8***

In a one-necked flask, a mixture of Ethyl 2-benzoyl-4-nitro-3-((S)-1,4-dioxaspiro[4.5]decan-2-yl)butanoate **7** (0.672 g, 1.66 mmol) in equal volumes of water and tetrahydrofuran was stirred. Trifluoroacetic acid (600 µL, 7.78 mmol) was then added and the reaction mixture was stirred at RT until the disappearance of the substrate. After the evaporation of the solvents under reduced pressure, the crude mixture was purified by flash column chromatography on silica gel (eluent: exhane/AcOEt = 7/3). The product was obtained as colorless liquid (0.175 g, 32%). The product is in the enol form, as suggested by the lack of one proton signal in the ^1^H NMR and the signal at 100.89 ppm in the ^13^C NMR spectra. ^1^H NMR (300 MHz, CDCl_3_) δ 7.78–7.70 (m, 2H), 7.53–7.35 (m, 3H), 4.98 (dd, J = 11.6, 5.8 Hz, 1H), 4.80 (dd, J = 9.8, 4.8 Hz, 1H), 4.58 (dd, J = 13.3, 9.8 Hz, 1H), 4.21–4.15 (m, 2H), 4.15–4.10 (m, 2H), 3.96 (m, 1H), 1.23 (t, J = 11.5 Hz, 3H). ^13^C NMR (75 MHz, CDCl_3_) δ 167.64, 164.11, 131.13, 129.50, 129.10, 127.71, 100.89, 85.32, 64.02, 60.46, 44.42, 29.68, 14.03. ESI-MS requires 325.1162, found 308.1134.

### 3.7. General Procedure for the Synthesis of 3-((R)-2-nitro-1-((S)-1,4-dioxaspiro[4.5]decan-2-yl)ethyl)pentane-2,4-dione ***10***

To a stirred solution of acetylacetone **9** (96.81 mL, 0.938 mmol) and Takemoto catalyst (19.39 mg, 10% mol, 0.469 mmol) in 1 mL of dry toluene was added nitro alkene **2** (100 mg, 0.469 mmol) at 35 °C. After being stirred for 24 h, the reaction mixture was concentrated in vacuo. Then the reaction crude was purified by flash column chromatography obtaining a yellow oil (hexane/EtOAc 4:1, 70% yield). ^1^H NMR (300 MHz, CDCl_3_) δ 4.61 (dd, J = 13.7, 5.8 Hz, 1H), 4.47 (dd, J = 13.7, 3.5 Hz, 1H), 4.09 (td, J = 13.7, 6.0 Hz, 2H), 3.64 (t, J = 6.9 Hz, 1H), 3.06 (s, 1H), 2.34 (s, 3H), 2.30 (s, 3H), 1.52 (t, J = 30.8 Hz, 10H); ^13^C NMR (75 MHz, CDCl_3_) δ 203.01, 202.09, 110.52, 74.95, 74.39, 67.54, 66.28, 40.87, 36.04, 34.65, 31.09, 30.08, 29.67, 24.95, 23.89, 23.71. ESI-MS requires 313.1525, found 313.1527.

### 3.8. General Procedure for the Synthesis of (5S)-methyl 5-(hydroxymethyl)-4-(nitromethyl)-2-oxotetrahydrofuran-3-carboxylate **12** A solution of Dimethyl 2-(2-nitro-1-((S)-1,4-dioxaspiro[4.5]decan-2-yl)ethyl)malonate ***5a**/**5b***

(0.239 g, 0.692 mmol) in TFA (230 µL, 3.00 mmol), 0.45 mL of H_2_O, and 0.45 mL of THF was stirred at RT for 3 h. Then the reaction mixture was evaporated under reduced pressure. The crude mixture was purified by column chromatography on silica gel (hexane/EtOAc = 3/7 as eluent). After evaporation of the solvent under reduced pressure, an orange oil is obtained (0.106 g, 65%). ^1^H NMR (300 MHz, CD_3_OD) δ 4.79 (dd, J = 9.2, 3.5 Hz, 2H), 4.40–4.34 (m, 1H), 3.86 (dd, J = 12.7, 3.3 Hz, 1H), 3.79 (s, 3H), 3.71 (dd, J = 12.7, 4.2 Hz, 1H), 3.65–3.55 (m, 1H); ^13^C NMR (75 MHz, CD_3_OD) δ 170.83, 167.75, 80.97, 74.67, 60.87, 52.06, 38.37; HRMS (ESI+) requires 233.0536, found 203.19810 [(M^+^-CH_2_OH)]. The compound was also fully characterized using CD_3_CN as a solvent; the J values showed a trans-trans configuration of the protons on the cycle (see Supplementary Materials). ^1^H NMR (500 MHz, CD_3_CN) δ 4.37 (ddd, J = 7.5, 4.0, 3.0 Hz, 1H), 3.85 (dd, J = 9.2, 3.6 Hz, 1H), 3.81 (d, J = 10.0 Hz, 1 H), 3.78 (s, 3H), 3.73 (dd, J = 8.6, 3.4 Hz, 2H) 3.68 (d, J = 9.9, 3.8 Hz, 1H), 3.6 (dd, J = 8.0, 7.5 Hz, 1H); ^13^C NMR (125 MHz, CD_3_CN) δ 170.30, 167.59, 80.57, 60.83, 52.67, 51.20, 50.17, 38.12

### 3.9. General Procedure for the Synthesis of (5S)-methyl 4-(aminomethyl)-5-(hydroxymethyl)-2-oxotetrahydrofuran-3-carboxylate ***13***

The 5-(*S*)-methyl 5-(hydroxymethyl)-4-(nitromethyl)-2-oxotetrahydrofuran-3-carboxylate **12** (0.175 g, 0.749 mmol) was placed into a beaker and dissolved in methanol (15 mL). The reaction mixture was pumped into ThalesNano H-Cube mini containing a cartridge of Pd/C (10%) with 1 mL/min as flow rate, at the pressure 15 mbar of H_2_ and at 35 °C. Reaction progress was monitored by TLC. After 3 h, the crude mixture was passed through a short silica pad, concentrated under vacuum, and then purified by flash column chromatography on silica gel (DCM/MeOH and 2% TEA = 9/1 as eluent) in order to afford the product (0.038 g, 22%). ^1^H NMR (300 Mhz, CD_3_OD) δ 4.48–4.44 (s, 1H), 3.86–3.81 (dd, 2H), 3.76–3.56 (dddd, 4H). ^13^C NMR (125 MHz, CD_3_OD) 172.01, 170.88, 85.77, 65.04, 62.03, 44.90, 38.59. HRMS (ESI+) requires 171.0532, found 140.03456 [(M^+^-CH_2_OH)].

## 4. Conclusions

Carbohydrates represent a widely available, chiral, renewable resource. They are a biomass-derived, highly diversified platform that could be employed to develop new sustainable synthetic pathways. Among the hexoses and the pentoses present in the biomass, in the present project, mannitol was selected as a valuable starting material, directly available from the chiral pool, to build highly functionalized molecules. Starting from (*R*)-2,3-O-Cyclohexylidene glyceraldehyde, easily prepared on large scale from D-mannitol, by reaction with nitromethane, an enantiopure chiral nitro alkene was prepared and its reactivity explored [22]. Organocatalytic Michael addition of dimethyl malonate or β-ketoesters on the nitro alkene afforded high stereoselectivity and densely functionalized chiral molecules, which were further synthetically elaborated, leading to five-membered lactones and bicyclic lactams [23]. Preliminary studies showed that the metal-free catalytic reaction on the chiral nitro alkene can also be performed under continuous flow conditions, thus enabling the use of (micro)mesofluidic systems for the preparation of enantiomerically pure organic molecules from the chiral pool [24].

## Supplementary Materials

The following are available online at https://www.mdpi.com/1420-3049/24/24/4588/s1. Figures S1–S8: NMR spectra of compounds **5a**/**5b**, **12**, **13**.
