# Supplementary material for: Organocatalytic Michael Addition to (D)-Mannitol-Derived Enantiopure Nitroalkenes: A Valuable Strategy for the Synthesis of Densely Functionalized Chiral Molecules
Source: Molecules. 2019 Dec 14;24(24):4588. doi: 10.3390/molecules24244588 (PMC6943540; doi:10.3390/molecules24244588)
Supplement: Supplementary file 1 [file molecules-24-04588-s001.pdf]

Supplementary Material

Organocatalytic Michael Addition to (D)-Mannitol-Derived Enantiopure Nitroalkenes: A Valuable Strategy for the Synthesis of Densely Functionalized Chiral Molecules.

Lucia Caruso, Alessandra Puglisi, Emmerance Gillon and Maurizio Benaglia *

Dipartimento di Chimica, Università degli Studi di Milano, Via Golgi 19, 20133 Milano, Italy; lucia.caruso@unimi.it (L.C.); alessandra.puglisi@unimi.it (A.P.); emmerance.gillon@student.uclouvain.be (E.G.)

* Correspondence: Maurizio.benaglia@unimi.it; 02 5031 4171 (M.B.).

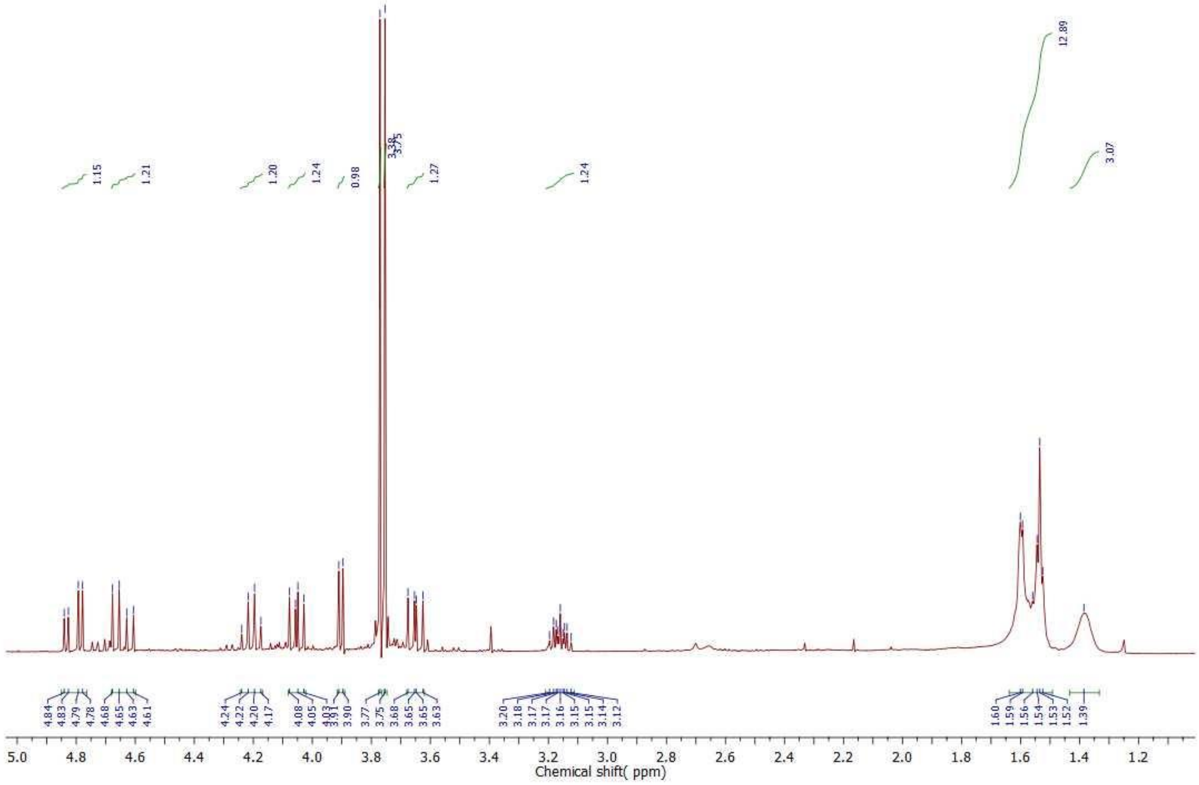

Figure **S1**, ^{1}H-NMR (300 Mhz, $CDCl_3$) Compounds **5a**/**5b** for reaction with *S*,*S*-Takemoto catalyst

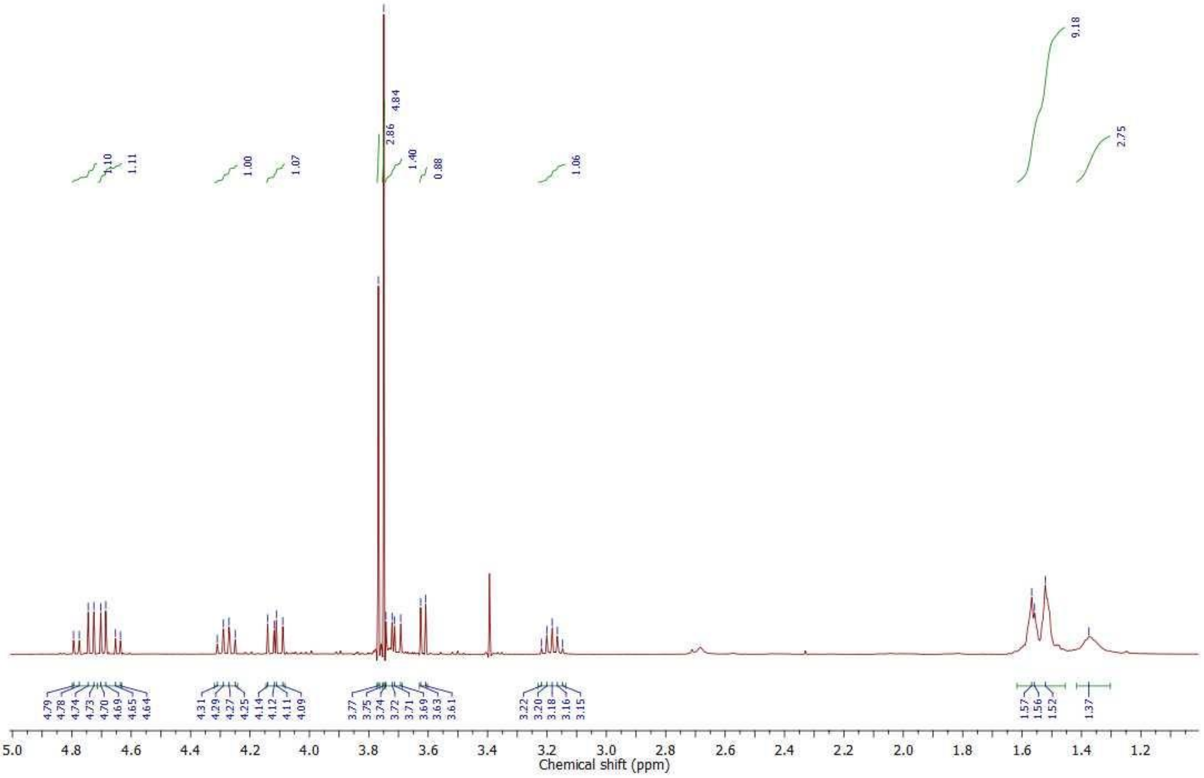

Figure **S2**, ^{1}H-NMR (300 Mhz, $CDCl_3$) Compounds **5b**/**5a** for reaction with *R*,*R*-Takemoto catalyst

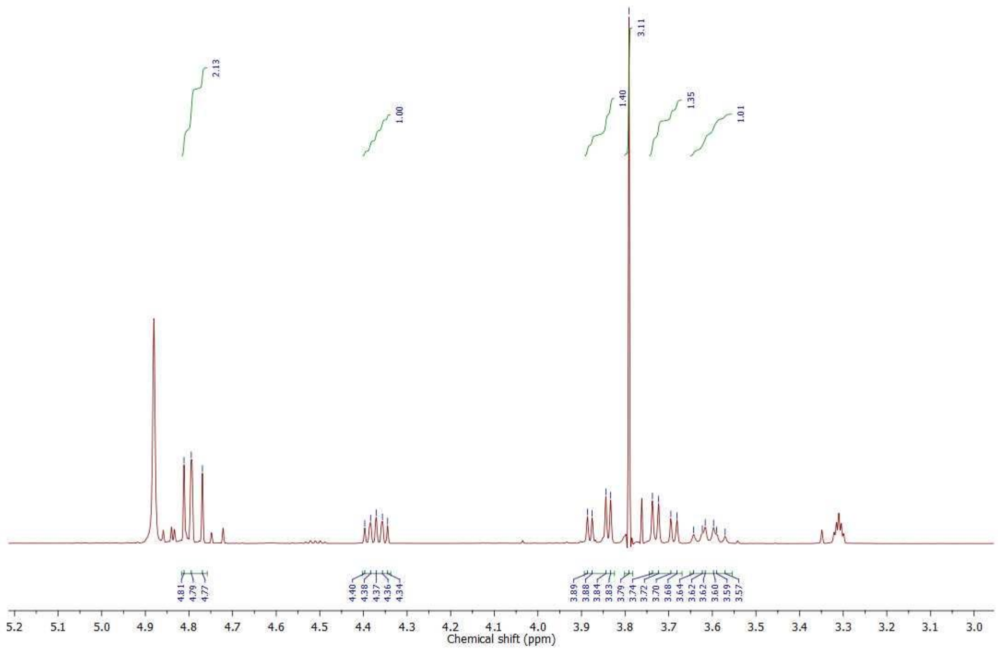

Figure **S3**, ¹H-NMR (300 Mhz, CD_3OD) Compound **12** for reaction with *S*,*S*-Takemoto catalyst

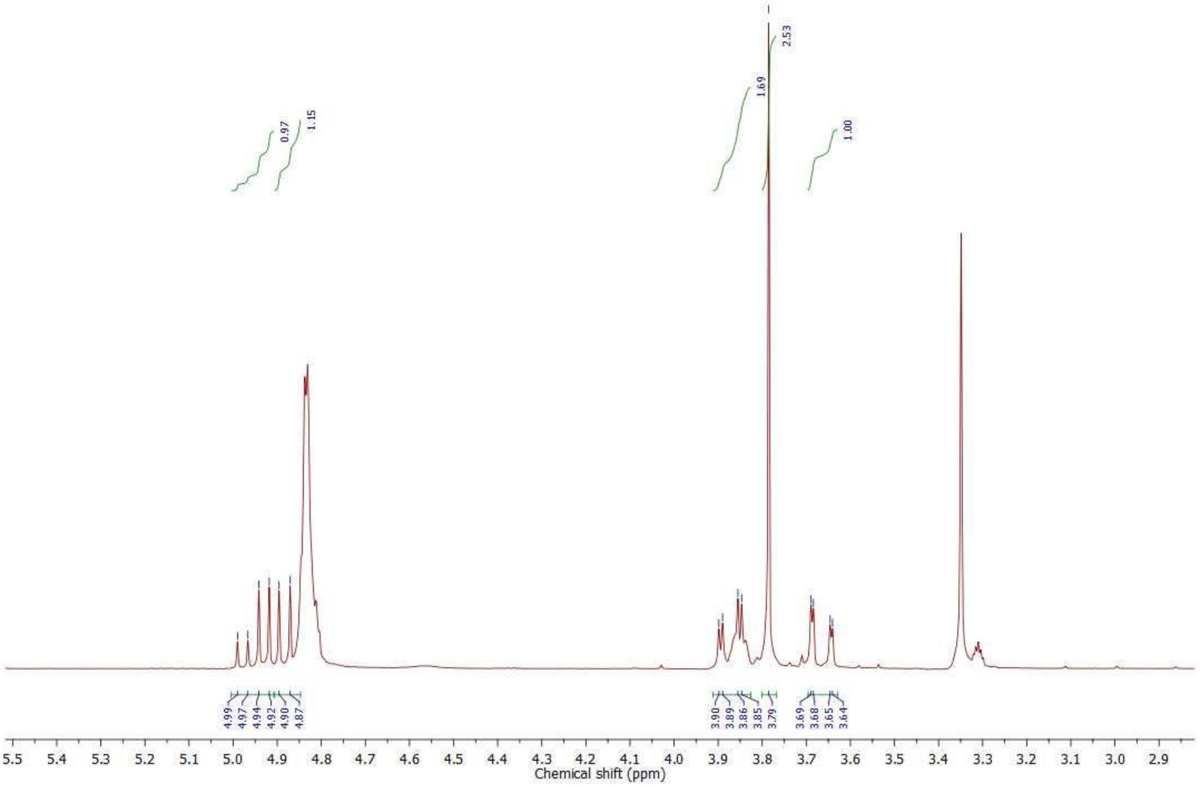

Figure **S4**, ¹H-NMR (300 Mhz, CD_3OD) Compound **12** for reaction with *R*,*R*-Takemoto catalyst

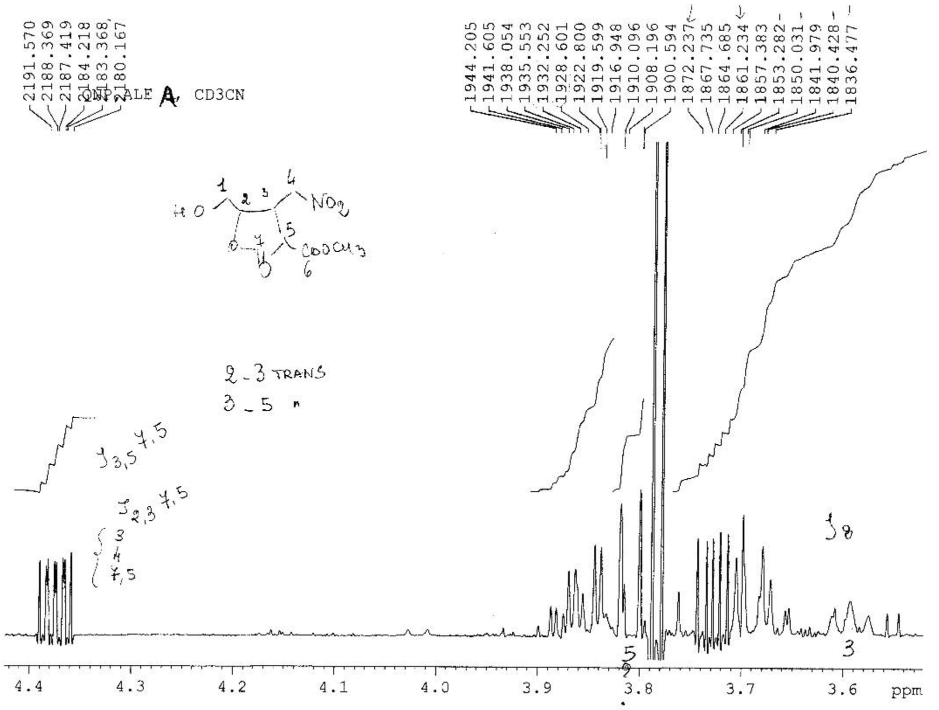

Figure **S5**, ^{1}H-NMR (300 Mhz, CD_3CN) Compound **12**

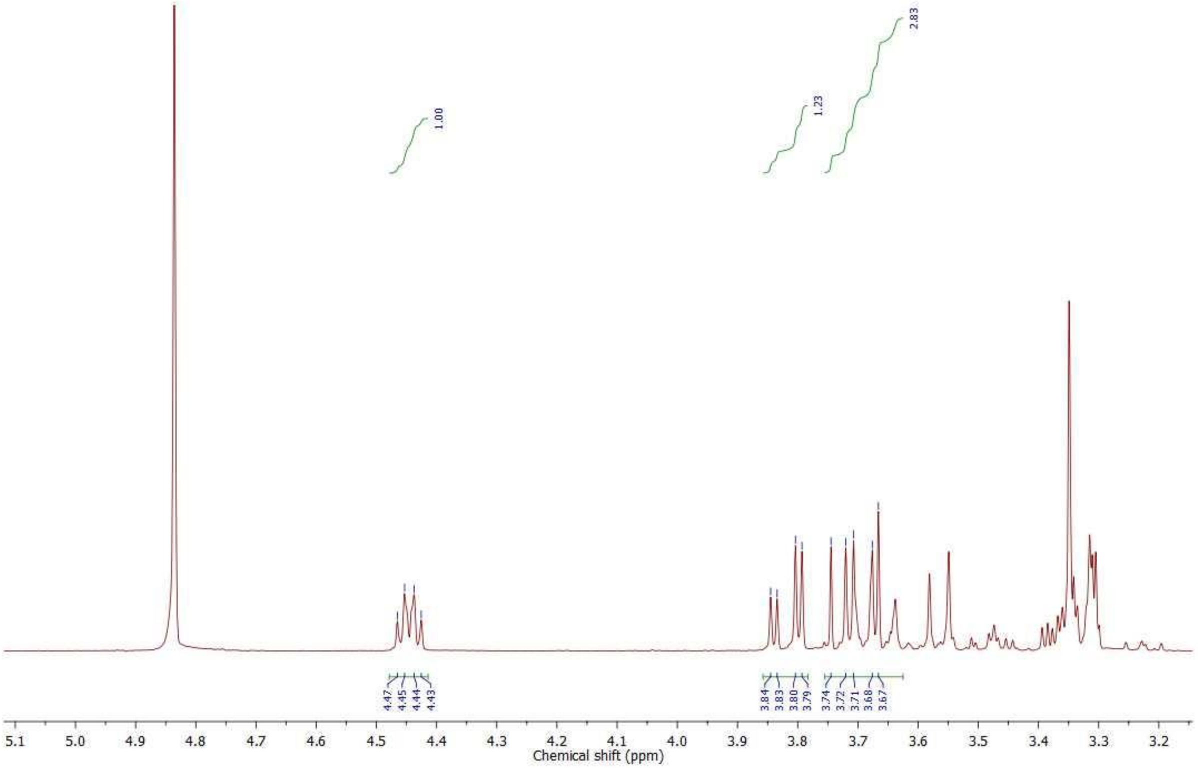

Figure **S6**, ^{1}H-NMR (300 Mhz, CH_3OD) Compound **13**

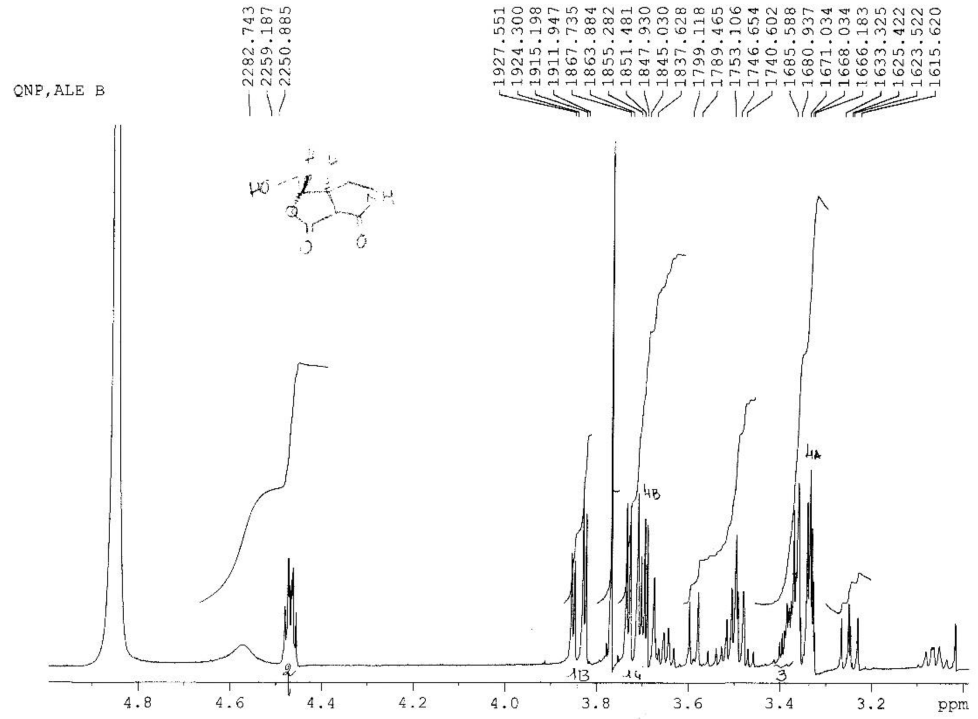

Figure **S7**, ^{1}H-NMR (500 Mhz, CD_3OD) Compound **13**

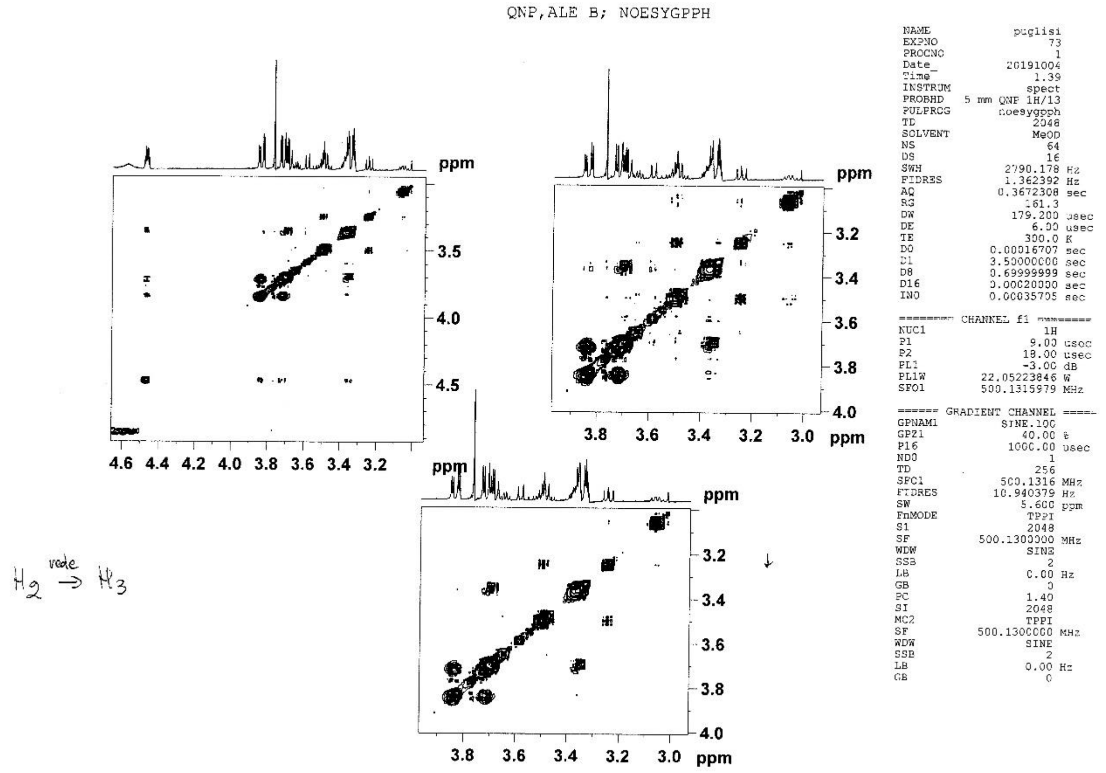

Figure **S8**, NOESY (500 Mhz, CD_3OD) Compound **13**
